# Brewers’ rice induces apoptosis in azoxymethane-induced colon carcinogenesis in rats via suppression of cell proliferation and the Wnt signaling pathway

**DOI:** 10.1186/1472-6882-14-304

**Published:** 2014-08-16

**Authors:** Bee Ling Tan, Norhaizan Mohd Esa, Heshu Sulaiman Rahman, Hazilawati Hamzah, Roselina Karim

**Affiliations:** Department of Nutrition and Dietetics, Faculty of Medicine and Health Sciences, Universiti Putra Malaysia, 43400 Serdang, Selangor Malaysia; Laboratory of Molecular Biomedicine, Institute of Bioscience, Universiti Putra Malaysia, 43400 Serdang, Selangor Malaysia; Department of Veterinary Pathology and Microbiology, Faculty of Veterinary Medicine, Universiti Putra Malaysia, 43400 Serdang, Selangor Malaysia; Department of Food Technology, Faculty of Food Science and Technology, Universiti Putra Malaysia, 43400 Serdang, Selangor Malaysia

**Keywords:** Brewers’ rice, Colon cancer, Beta-catenin, Ki-67, Apoptosis

## Abstract

**Background:**

Brewers’ rice is locally known as *temukut*, is a byproduct of the rice milling process, and consists of broken rice, rice bran, and rice germ. Unlike rice bran, the health benefit of brewers’ rice has yet to be fully studied. Our present study aimed to identify the chemopreventive potential of brewers’ rice with colonic tumor formation and to examine further the mechanistic action of brewers’ rice during colon carcinogenesis.

**Methods:**

Male Sprague–Dawley rats were randomly divided into five groups: (G1) normal; (G2) azoxymethane (AOM) alone; and (G3), (G4), and (G5), which were AOM fed with 10%, 20%, and 40% (w/w) of brewers’ rice, respectively. Rats in group 2 to 5 were injected intraperitoneally with AOM (15 mg/kg body weight) once weekly for two weeks. Colon tumor incidence and multiplicity was assessed by hematoxylin and eosin (H&E) staining. The expression of β-catenin, cyclooxygenase-2 (COX-2), and Ki-67 was evaluated by immunohistochemical staining. The apoptosis-inducing activity was analyzed using a TUNEL assay. The data were analyzed using a one-way analysis of variance (ANOVA) with P-value<0.05 was considered significant.

**Results:**

Overall analyses revealed that brewers’ rice reduced colon tumor incidence and multiplicity. The results from immunohistochemistry analysis also showed that brewers’ rice decreased the expression of β-catenin, COX-2, and Ki-67 in a dose-dependent manner. Furthermore, TUNEL analysis demonstrated that administration of brewers’ rice in AOM-induced rat colorectal cancer resulted in a dose-dependent increase in cell apoptosis.

**Conclusions:**

Taken together, our data suggested that brewers’ rice can inhibit cell proliferation, induce apoptosis, and suppress COX-2 and β-catenin expression via the Wnt signaling pathway and holds great promise in the field of chemoprevention as a dietary agent.

## Background

Colorectal cancer has become a significant global health concern and represents the fourth leading cancer in males and the third most common cancer in females worldwide [1]. According to the National Cancer Registry Malaysia [2], colorectal cancer has become the second most common cancer following breast cancer, being first among males and second among females in Peninsular Malaysia in 2006. Generally, activity of the Wnt/β-catenin signaling pathway is dependent on the level of β-catenin in the cytoplasm. Overexpression of cyclooxygenase-2 (COX-2) and uncontrolled wingless and Wnt signaling pathway have been reported to contribute to colorectal cancer [3]. In most normal tissues, COX-2 expression is undetectable; however, during an inflammatory reaction, it can be rapidly induced [4, 5]. Ki-67 is closely correlated with somatic cell proliferation [6]. Increasing colon epithelial cell proliferation has been characterized as hyperplasia, which can be identified using the Ki-67 proliferation marker [7].

Whole grain consumption has been demonstrated to be protective against colorectal cancer in human intervention studies [8]. Rice by-products have received increased attention as functional foods due to their phenolic base compounds, as well as having high levels of vitamins, minerals, and fiber contents [9]. Brewers’ rice is locally known as *temukut*, is a by-product of the rice milling process, and consists of broken rice, rice bran, and rice germ. Previous findings have demonstrated that rice bran inhibits human colon cancer cell growth [10], and consumption of rice bran has also been reported to reduce the number of intestinal adenomas in *APC*^*Min*^ mice [11]. Several studies as reported by Norhaizan et al. [12] also shown that rice germ can prevent AOM-induced colonic aberrant crypt foci (ACF) in rats. Unlike rice bran, the health benefit of brewers’ rice has yet to be fully studied. Because our earlier study demonstrated that water extract of brewers’ rice was cytotoxic against a colorectal cancer (HT-29) cell line with IC_50_ 38.33 μg/mL [13], we further investigated the chemopreventive potential of brewers’ rice and colonic tumor formation as the endpoint and also investigated whether brewers’ rice confers inhibitory effects via the expression of Ki-67 and the rate of apoptosis. Moreover, the effects of brewers’ rice on the expression of β-catenin and COX-2 during colon carcinogenesis were also assessed.

We found brewers’ rice reduced colon tumor incidence and multiplicity. The results from immunohistochemistry analysis also revealed that brewers’ rice decreased the expression of β-catenin, COX-2, and Ki-67 in a dose-dependent manner. In addition, TUNEL analysis showed that administration of brewers’ rice in AOM-induced rat colorectal cancer resulted in a dose-dependent increase in apoptotic cell.

## Methods

### Chemicals and reagents

AOM and 10% (v/v) neutral buffered formalin were purchased from Sigma (St. Louis, MO, USA). The DeadEnd™ Fluorometric TUNEL System was purchased from Promega (Madison, WI, USA). Dako REAL™ EnVision™ Detection System was purchased from DAKO (Carpinteria, CA, USA). All other chemicals and reagents used were of analytical grade and purchased from Sigma-Aldrich (St. Louis, MO, USA).

### Brewers’ rice

Freshly milled brewers’ rice sample (from rice variety MR 219) was obtained from a local milling factory, BERNAS at Seri Tiram Jaya, Selangor, Malaysia.

### Diet and animals

**Figure 1:**
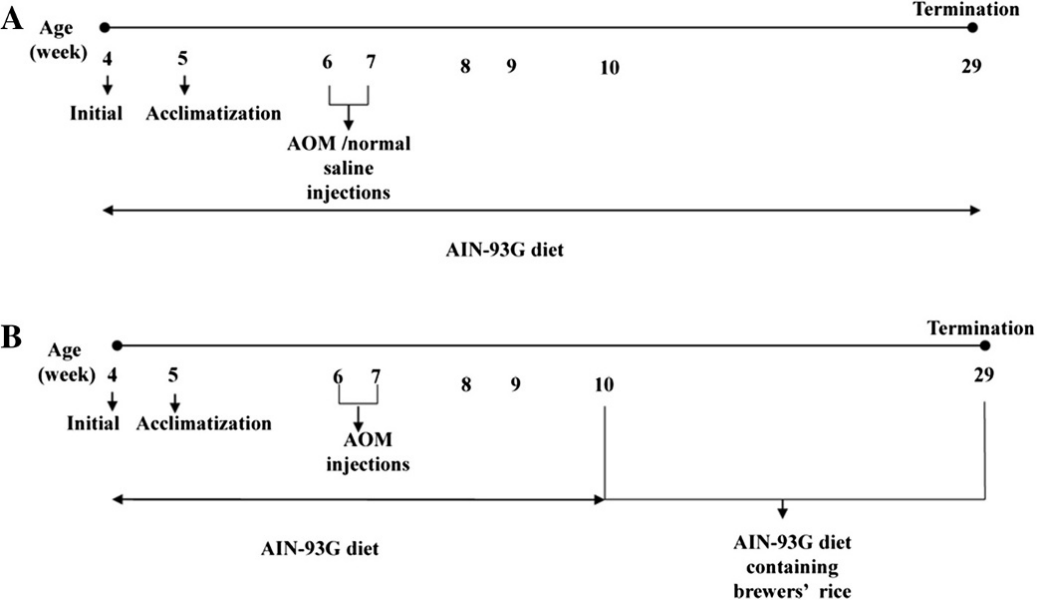
**Experimental design of the study. (A)** Time line of experiment for Group 1 and Group 2 **(B)** Time line of experiment for Group 3, 4, and 5.

Stabilization of brewers’ rice has been described in our previous study [13]. This study was performed according to the guidelines approved by the Animal Care and Use Committee (ACUC) of the Faculty of Medicine and Health Sciences, Universiti Putra Malaysia (UPM) Serdang, Selangor with approval number UPM/FPSK/PADS/BR-UUH/00461. A total of 30 four-week-old male Sprague–Dawley rats (*Rattus Norwegicus*), weighing approximately 90–100 grams were housed in plastic cages (two rats per cage) with wood-chip bedding. The animals were acclimatized for 1 week and fed with an American Institute of Nutrition (AIN-93G) diet *ad libitum*. They were housed in a well-ventilated room at approximately 25 to 27°C, 50 ± 10% relative humidity, and a 12-hour light/dark cycle. Hygienic conditions were maintained by weekly changes of woodchip beds. The rats were randomly divided into five groups (n = 6 rats for each group), including (G1): normal, (G2): AOM alone, (G3): AOM + 10% (w/w) of brewers’ rice, (G4): AOM + 20% (w/w) of brewers’ rice, and (G5): AOM + 40% (w/w) of brewers’ rice. At six weeks of age, animals from G2 to G5 were induced with AOM intraperitoneally (15 mg/kg body weight) once weekly for two weeks, whereas the rats in the normal group received an equal volume of normal saline and served as the vehicle control. The control group (G1 and G2) received an AIN-93G diet. G3, G4, and G5 received an AIN-93G diet containing 10%, 20%, and 40% (w/w) of brewers’ rice, respectively (Table 1). The compositions of the AIN-93G diet were adjusted according to the nutrient composition of brewers’ rice with moisture (11.36 ± 0.12%), ash (1.56 ± 0.26%), protein (9.01 ± 0.27%), fat (1.95 ± 0.11%), total available carbohydrate (72.42 ± 1.25%), and total dietary fiber (5.32 ± 0.04%) (unpublished observations, Tan Bee Ling) with the final percentages of carbohydrate, fat, and protein as 62.95%, 7%, and 20%, respectively. Body weight was recorded weekly throughout the study. Experimental design of the study is shown in Figure 1.

**Table 1:** **Composition of experimental diets**

Ingredients (g/1000 g diet)	Group				
	G1	G2	G3	G4	G5
Brewers’ rice	-	-	100.0	200.0	400.0
Corn starch	397.5	397.5	315.3	233.2	68.9
Casein	200.0	200.0	191.0	182.0	164.0
Maltodextrin	132.0	132.0	132.0	132.0	132.0
Sucrose	100.0	100.0	100.0	100.0	100.0
Soybean oil	70.0	70.0	68.1	66.1	62.2
Powdered cellulose	50.0	50.0	44.7	39.4	28.7
AIN-93G mineral mix	35.0	35.0	33.4	31.9	28.8
AIN-93G vitamin mix	10.0	10.0	10.0	10.0	10.0
L-cystine	3.0	3.0	3.0	3.0	3.0
Choline bitartrate	2.5	2.5	2.5	2.5	2.5
tert-butylhydroquinone	0.014	0.014	0.014	0.014	0.014

G1 and G2, AIN-93G diet; G3, AIN-93G diet containing 10% (w/w) of brewers’ rice; G4, AIN-93G diet containing 20% (w/w) of brewers’ rice; G5, AIN-93G diet containing 40% (w/w) of brewers’ rice.

### Carcinogen injection

Azoxymethane (AOM), a specific carcinogen was diluted in 0.9% (v/v) saline and the animals were injected intraperitoneally once weekly (15 mg/kg body weight) for 2 weeks to induce colonic tumors [14].

### Tumor assessment

After twenty weeks of treatment, the rats were sacrificed after anesthesia with diethyl ether. Colon tissue was removed, dissected longitudinally, flushed with phosphate-buffered saline (PBS), fixed in 10% (v/v) neutral buffered formalin prior to staining with hematoxylin and eosin (H&E). The tumor incidence was assessed according to Bird et al. [15] and Rao et al. [16]. The tumor incidence was described as the percentage of total animals with adenoma/adenocarcinoma while tumor multiplicity was defined as the average number of tumors per tumor-bearing rat. The number and size of the colon tumors present was also recorded.

### Immunohistochemical staining of β-catenin, COX-2, and Ki-67 antigen

Tissue sections from the paraffin embedded tissue block was cut into 4–6 μm thickness using a Leica rotation microtome and affixed onto 3-aminopropyltrimethoxysilane (APES)-treated slides. The paraffin embedded tissue slides were heated in an oven at 60°C for 1 hour. The tissue sections were deparaffinized and rehydrated by sequential immersion in xylene two times, and a graded ethanol series (100%, 90%, 70%, and 50% (v/v)) for 3 minutes each at room temperature, followed by a rinse in slow running tap water at least three times. Sections were demasked by immersed in boiling sodium citrate buffer (pH 6.0) for 5 minutes in a microwave oven to expose the antigenic sites that might be hindered during the embedding process and to permeabilize the cells to antibodies. The sections were rinsed with distilled water and washed two times with phosphate-buffered saline in 0.1% Tween-20 (PBST) for 3 minutes each before incubation with 3% (v/v) of hydrogen peroxide (H_2_O_2_) for 10 minutes at room temperature to quench endogenous peroxide. After rinsing with PBST two times and distilled water for 3 minutes each, the sections were incubated with primary antibody overnight at 4°C. The primary antibodies included β-catenin (E-5) mouse monoclonal antibody (1:50 dilution, catalogue number sc-7963; Santa Cruz Biotechnology, Inc.), COX-2 (N-20) antibody (1:50 dilution, catalogue number sc-1746; Santa Cruz Biotechnology, Inc.), and rabbit polyclonal to Ki-67 (1:200 dilution, catalogue number AB 66155, Abcam). All antibodies were diluted with antibody diluent prior to use. Immunostaining was performed using the Dako REAL™ EnVision™ Detection System according to the manufacturer’s instructions. After rinsing with PBST two times and distilled water for 3 minutes each, 500 μL ready-made secondary antibody was applied and incubated for 1 hour at room temperature. All steps were followed by rinsing with PBST two times and distilled water for 3 minutes each. Peroxidase activities were detected with 300 μL of 3, 3’Diaminobenzidine (DAB) chromogen substrate for 10 minutes at room temperature resulting in the presence of a brown precipitate. The tissue sections were counterstained with 500 μL hematoxylin for 3 minutes. Dehydration was performed by incubation of the tissue sections in xylene, 50%, 70%, 90%, and 100% ethanol for 3 minutes each and mounted. After drying, the slides were observed under a light microscope.

### Evaluation of immunohistochemical staining

A semi-quantitative scoring system was adopted according to Kohno et al. [17] to evaluate antibody staining against β-catenin, COX-2, and Ki-67. The total score was evaluated by the sum of the extent and intensity of the staining (score = extent + intensity) of seven fields in sections that were stained with the respective antibody using a 100× magnification selected from each section of the colon per rat. The percentage of positive cells was examined using the following scale: 0 = no staining of cells in any field; 1 = positive staining in 1 to 25%; 2 = positive staining in 26 to 50%; 3 = positive staining in 51 to 75%; and 4 = positive staining in 76 to 100%. The strength staining intensity was evaluated using the following range: 0, no staining of cells; 1+, mild staining; 2+, moderate staining, and 3+, strong staining. Thus, the maximum score was 7 and the minimum score was 0 after summation.

### Apoptosis assay

The TdT-mediated dUTP Nick-End Labeling (TUNEL) assay was performed using the DeadEnd™ Fluorometric TUNEL System for specific detection and quantification of apoptotic cells, according to the manufacturer’s protocols. Tissue sections from the paraffin embedded tissue block was cut into 4–6 μm thickness using a Leica rotation microtome and affixed onto the APES-treated slides. The tissue sections were deparaffinized and rehydrated by sequential immersion in xylene two times, and a graded ethanol series (100%, 90%, 70%, and 50% (v/v)) for 3 minutes each at room temperature followed by 0.85% of sodium chloride for 5 minutes. The tissue section was immersed in 1% formaldehyde, proteinase K, and 1% formaldehyde for 15, 10, and 5 minutes, respectively. All steps were followed by rinsing with PBS for 5 minutes each. The tissue section was then immersed in equilibration buffer for 10 minutes. Before incubation at 37°C for 1 hour, Terminal DeoxynucleotidylTransferase, Recombinant, enzyme (rTdT) was added and covered with a plastic coverslip. The slides were immersed in 2 × SSC for 15 minutes. The tissue sections were counterstained with propidium iodide (PI) for 15 minutes followed by rinsing with deionized water for 5 minutes and mounted. After drying, the slides were observed under a fluorescence microscope with 400× magnification. Colon tissue sections with FITC-positive cells were scored as apoptotic cells (green fluorescent cells). The total number of apoptotic cells was quantified in four randomly selected microscopic fields at 400× magnification and the percentage of apoptotic cells was defined as the number of FITC-positive cells per field/total number of cells counted per microscopic field × 100%.

### Statistical analysis

Statistical analyses were performed according to the Statistical Package for Social Science (SPSS) version 17.0. The data were expressed as the mean ± standard deviation (SD) and analyzed using a one-way analysis of variance (ANOVA). A P-value<0.05 was considered significant.

## Results and discussion

### Body weight

**Figure 2:**
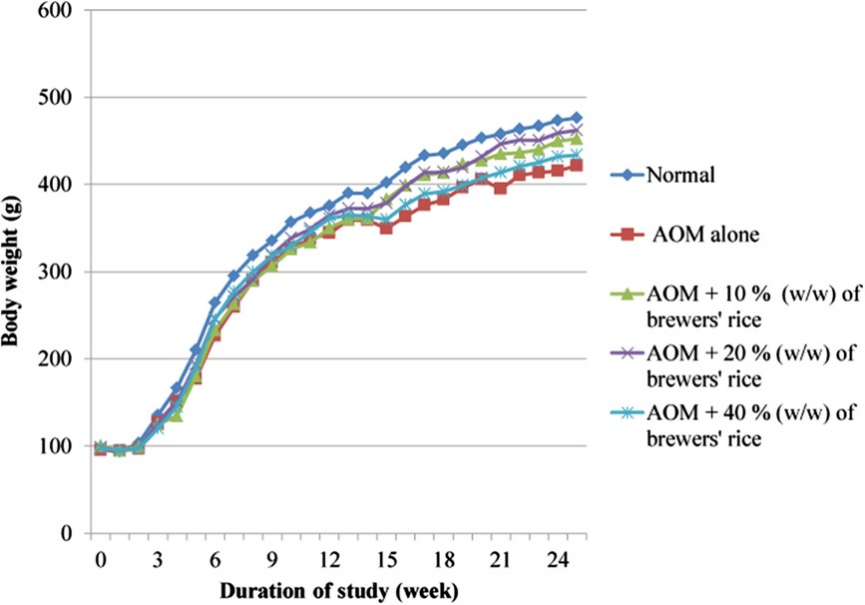
**Changes in body weight of experimental rats.**

The changes in body weight throughout the experiment are summarized in Figure 2. The body weight of the rats was compared among all groups throughout the study: [normal, AOM alone, and brewers’ rice-fed groups (AOM + 10%, 20%, and 40% (w/w) of brewers’ rice). Expectedly, the body weight of AOM-treated rats was lower compared to all those animals fed with diets containing brewers’ rice at the termination of the study. However, the body weight gain for all brewers’ rice-fed groups was nearly similar to the normal group and did not show any significant difference (P>0.05). The absence of significant differences in body weight in the present study demonstrated that dietary intake had no apparent influence on the results. Furthermore, because the rats consuming the brewers’ rice diet were able to thrive and gain weight in a manner similar to that of the rats fed the control diet, this finding indicates that the highest dosage of brewers’ rice diet (up to 40% (w/w)) was well-tolerated by rats.

### Number and size of colon tumors

**Figure 3:**
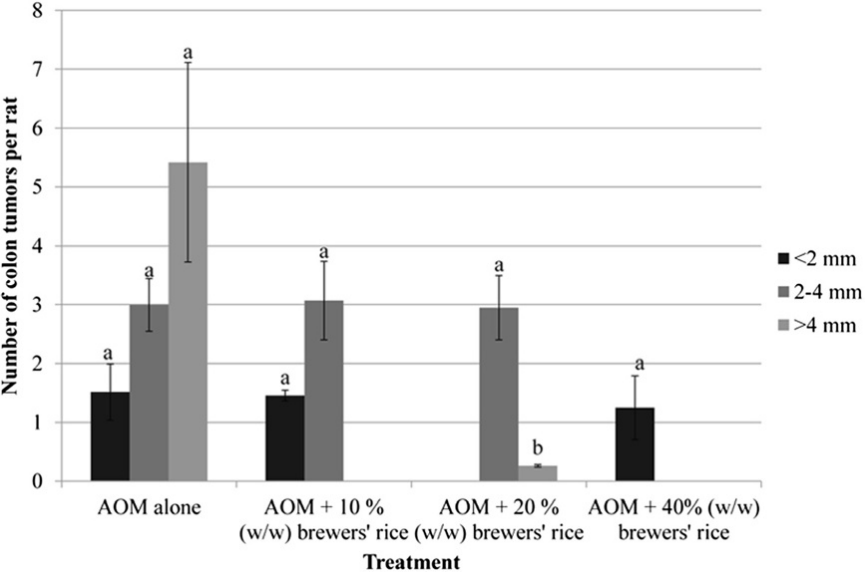
**Effect of brewers’ rice treatment on the size of colon tumors.**

As shown in Figure 3, the highest number of colon tumors was found in the AOM alone. After twenty weeks of treatment with brewers’ rice, the number of colon tumors was reduced. No significant difference was observed in colon tumor sizes less than 2 mm in diameter among the AOM-alone group, 10%, and 40% (w/w) brewers’ rice (P>0.05). A similar trend (no significant difference) was also found in colon tumors size 2–4 mm diameter in the AOM-alone group, 10%, and 20% (w/w) brewers’ rice (P>0.05). However, the suppressive effect of brewers’ rice on colon tumor sizes greater than 4 mm in diameter was notable in brewers’ rice-fed group compared to the AOM-alone group (Figure 3).

### Brewers’ rice reduces tumor incidence and tumor multiplicity in AOM-injected rats

To explore whether dietary administration of brewers’ rice on AOM-induced colon tumorigenesis mediated the retardation of tumor incidence and tumor multiplicity development, Sprague–Dawley rats were administered with different doses (10%, 20%, and 40% (w/w)) of brewers’ rice. A dosage of 10% (w/w) of brewers’ rice was selected as recommended in an earlier study by Boateng et al. [18] on rice bran and rice germ. This concentration has been demonstrated to inhibit tumor incidence. Furthermore, a higher concentration (20% and 40% (w/w) brewers’ rice) was used in this present study to evaluate the dose-dependent effect of brewers’ rice as a chemopreventive agent in a rat colon cancer experimental model.

**Figure 4:**
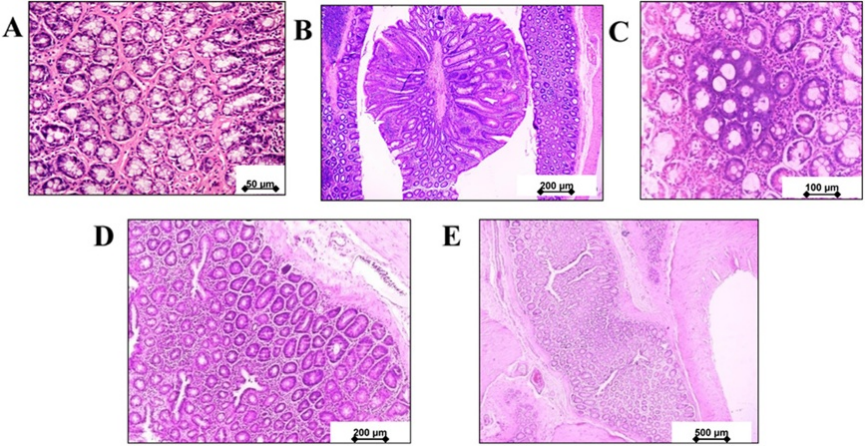
**Histopathology of colonic lesions developed in rats group treated with AOM.** (a) Adenomatous polyps originated from the colon of few rats **(B-C)** (b) Adenocarcinoma **(D-E)** showing the invasive gland in the submucosa and muscular layer. (**(A)** Magnification 400x), (**(B)** and **(D)** Magnification 100x), (**(C)** Magnification 200x), (**(E)** Magnification 40x).

Adenoma is defined as a benign colonic tumor, which consists of the proliferation of the colonic gland lined with neoplastic colonic epithelium, whereas adenocarcinoma is a malignant colonic tumor consisting of invasive glands lined with pleomorphic hyperchromatic epithelium. None of the rats in the normal group (without AOM injection) developed tumors when autopsied after 20 weeks of treatment (Table 2 and Figure 4). At the termination of the study, the AOM alone rats and 40% (w/w) brewers’ rice-treated rats had a tumor incidence of 100% and 33%, respectively.

**Table 2:** **Tumor assessment of brewers’ rice-treated AOM-induced colon cancer**

Group no	Treatment	Incidence (% animals with adenoma and/or adenocarcinoma)			Tumor multiplicity (no. of tumors/rats)		
		Adenoma	Adenocarcinoma	Total	Adenoma	Adenocarcinoma	Total
1	Normal	0	0	0	0	0	0
2	AOM alone	6/6 (100%)	5/6 (83%)	6/6 (100%)	3.05 ± 0.81^a^	3.73 ± 0.70^b^	6.78 ± 0.48^b^
3	AOM + 10% (w/w) of brewers’ rice	4/6 (67%)	4/6 (67%)	4/6 (67%)	2.72 ± 1.73^a^	1.58 ± 0.56^ab^	4.30 ± 0.81^ab^
4	AOM + 20% (w/w) of brewers’ rice	3/6 (50%)	3/6 (50%)	3/6 (50%)	1.75 ± 1.10^a^	1.50 ± 0.80^ab^	3.25 ± 0.18^ab^
5	AOM + 40% (w/w) of brewers’ rice	2/6 (33%)	2/6 (33%)	2/6 (33%)	1.10 ± 0.59^a^	0.45 ± 0.38^a^	1.55 ± 0.46^a^

Each value expressed as mean ± SD. Value in the same column with different superscript letter indicates significant difference by Tukey test (P<0.05).

Administration of 40% (w/w) of brewers’ rice significantly reduced tumor multiplicity compared to the AOM-alone group (P<0.05). However, the colon tumor multiplicity did not significantly differ between the group fed with diets containing 10% and 20% (w/w) of brewers’ rice (P>0.05).

Histopathological analysis using H&E staining revealed that the highest incidence of adenoma, adenocarcinoma, and total tumor were present in the AOM-alone group compared to those fed with diets containing brewers’ rice. The colon tumor multiplicity did not significantly differ between the AOM-alone group and the group fed with diets containing 10% (w/w) brewers’ rice, between the AOM-alone group and 20% (w/w) brewers’ rice (P>0.05). The moderate reduction in tumor incidence and tumor multiplicity may be due to a low dose (10% (w/w) of brewers’ rice) used. However, administration of 40% (w/w) of brewers’ rice significantly reduced tumor multiplicity compared to the AOM-alone group (P<0.05). This finding indicated that the colon tumor incidence and tumor multiplicity was reduced in a dose-dependent manner after the administration of brewers’ rice in AOM-induced rat colon carcinogenesis. A study reported that nearly 90% of the colon cancer tumor occurred due to stimulation of mutations in the Wnt pathway [19]. In addition to excessive β-catenin expression observed during the early sequence of events, which underlie the colon carcinogenesis, COX-2 overexpression also contributed to the development of colon cancer, as reported by Spychalskiet al. [20]. Thus, the expression of β-catenin and COX-2 in response to brewers’ rice was analyzed in colon tissue.

### Treatment of brewers’ rice suppresses β-catenin expression in colonic tumors

The key effector that regulates the Wnt signaling pathway is β-catenin, where β-catenin in the cytoplasm is targeted for destruction via phosphorylation and degradation via the ubiquitin-proteasome pathway, whereas dephosphorylation promoted the accumulation of β-catenin in the cytoplasm, thereby resulting in the translocation of β-catenin into the nucleus. In the nucleus, β-catenin binds to the transcription factor lymphocyte enhancer factor (LEF)-1/T-cell factor (TCF) family to activate the transcription of Wnt target genes [21]. In brewers’ rice-treated AOM-induced rats, it was evident that phosphorylation of β-catenin was increased in a dose-dependent manner. As shown in Table 3, 10%, 20%, and 40% (w/w) brewers’ rice-treated AOM-induced rats expressed β-catenin at 102.60, 98.82, and 64.72, respectively.

**Table 3:** **Total score for the expression of β-catenin, COX-2, and Ki-67 in colonic tissue**

Groups	Normal	AOM alone	AOM + 10% (w/w) of brewers’ rice	AOM + 20% (w/w) of brewers’ rice	AOM + 40% (w/w) of brewers’ rice	p
Beta-catenin	0	115.05 ± 3.06^c^	102.60 ± 1.00^bc^	98.82 ± 0.58^b^	64.72 ± 1.53^a^	0
COX-2	49.03 ± 4.93^a^	123.45 ± 2.65^b^	117.84 ± 5.57^b^	106.32 ± 4.93^b^	95.47 ± 1.53^b^	0
Ki-67	0	107.42 ± 4.51^c^	95.41 ± 6.51^bc^	54.31 ± 4.36^ab^	43.12 ± 5.69^a^	0.003

Each value expressed as mean ± SD of three determinations. Value in the same row with different superscript letter indicates significant difference by Tukey test (P<0.05). Administration of 20% and 40% (w/w) of brewers’ rice significantly reduced β-catenin expression compared to the AOM-alone group (P<0.05). However, the COX-2 expression did not significantly differ between the AOM-alone group and the brewers’ rice-fed groups (P>0.05). In Ki-67, administration of 20% and 40% (w/w) of brewers’ rice significantly reduced in cell proliferation compared to the AOM-alone group (P<0.05).

**Figure 5:**
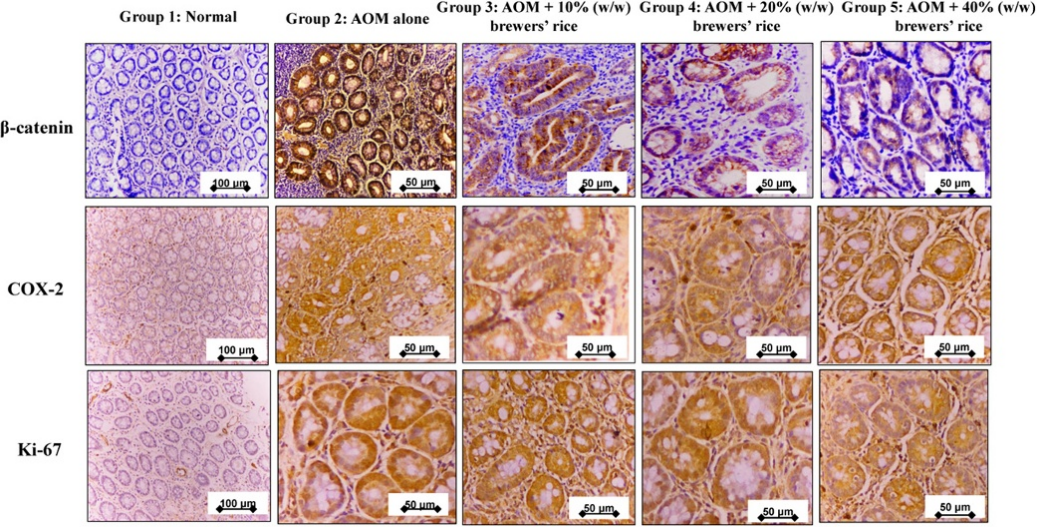
**Immunohistochemical staining of β-catenin, COX-2, and Ki-67.** The expression of β-catenin, COX-2, and Ki-67 in normal (Group 1), AOM alone (untreated) (Group 2) compared to treatment groups of rats with 10% (Group 3), 20% (Group 4), and 40% (Group 5) (w/w) of brewers’ rice (n = 3). Expression of β-catenin showed membrane, nucleus, and cytoplasmic staining: strong β-catenin in Group 2, weaker staining of β-catenin in Group 3 and Group 4, and weakest staining of β-catenin in Group 5. Expression of COX-2 in cytoplasm of colonic cells: strong COX-2 in Group 2 and Group 3, weaker staining of COX-2 in Group 4 and Group 5, and weakest staining of COX-2 in Group 1. Immunoreactivity of Ki-67 in colonic sections showed a sequence reduction of proliferative activity of the nucleus (Group 3, 4, and 5). Beta-catenin, COX-2, and Ki-67 in Group 1 (Magnification 200x), β-catenin, COX-2, and Ki-67 in Group 2, Group 3, Group 4, and Group 5 (Magnification 400x).

Expectedly, we found no localization of the signal on the cell membrane, cytoplasm, and nucleus of the epithelial cells and goblet cells in normal colon mucosa. This finding may indicate that the β-catenin level in normal colon mucosa was too low to be detected using immunohistochemistry (IHC). In the AOM-alone group (G2), carcinogen was induced but no treatment with brewers’ rice was provided, and intense β-catenin stain (immunoreactivity in the cytoplasm of epithelial cells is prominent) was observed and indicated much higher β-catenin levels. Immunohistochemical analysis revealed that the abundance of β-catenin was mostly present in the cytoplasm, and relatively high nuclear staining was observed in rats injected with AOM, whereas administration of brewers’ rice markedly reduced the expression of β-catenin in both the cytoplasm and the nucleus (Figure 5). The overall scoring demonstrated that the colon tissue of G2, which had the highest number of tumor incidence and tumor multiplicity among all groups (Table 2), exhibited the highest score in the evaluation of immunohistochemical staining of β-catenin compared to other treatment groups. The brown stain intensity was variable in which some of the cells showed a greater intensity at the cell membrane compared to the cytoplasm, while the other cells lost or demonstrated a weak brown color at the cell membrane, thus, suggesting higher localization of β-catenin expression in the compartment of the cytoplasm.

Heterogenous expression of β-catenin was also observed in the treatment groups (G3, G4, and G5) in the cell membrane and cytoplasm compartment, but only two treatment groups (20% and 40% (w/w) of brewers’ rice) demonstrated a significant reduction in β-catenin expression compared to G2 (P<0.05). However, no significant difference was observed between AOM alone and 10% (w/w) brewers’ rice (P>0.05) (Table 3). This finding indicated that brewers’ rice had the potential to reduce β-catenin expression. Thus, it was speculated that brewers’ rice might represent a natural chemopreventive agent that acts via the Wnt pathway which involves β-catenin. Because brewers’ rice suppresses β-catenin expression, the susceptibility of colon tumors to brewers’ rice might also be due to inhibition of the Wnt signaling pathway via the suppression of COX-2 expression. Thus, the expression of COX-2 in brewers’ rice during colon carcinogenesis was investigated to determine whether brewers’ rice can modulate this expression.

### Brewers’ rice inhibits the expression of COX-2 in colonic tumors

Overexpression of inflammatory markers is a hallmark in colorectal tumors [22] and COX-2 is an attractive target for colon cancer prevention and treatment. The aim of selective COX-2 inhibitor development such as valdecoxib, rofecoxib, and celecoxib is to prevent the side effects of non-steroidal anti-inflammatory drugs. It is well known that colon tumorigenesis can be inhibited using selective inhibition of COX-2. Although randomized clinical trials of selective COX-2 inhibitors against colon cancer, such as the Adenoma Prevention with Celecoxib trial and the Adenomatous Polyp Prevention on Vioxx trial demonstrated promising results; unfortunately, all trials were halted due to cardiovascular side effects [23]. Thus, the development of specific COX-2 inhibitors without cardiovascular disease risk is needed.

In addition to the effects on β-catenin, our current data revealed that colon tissue from the normal group (G1) exhibited a degree of COX-2 immunoreactivity in which COX-2 expression was observed to be weak, diffused, and heterogenous. This finding was consistent with the study reported by Kohno et al. [17], who demonstrated the COX-2 expression in normal colon mucosa. However, Kohno’s study contradicted with the study of Shao et al. [24]. The results showed that the AOM-alone group had the highest COX-2 expression with positive brownish staining of COX-2 predominantly localized in the cytoplasm. This finding was consistent with a study reported by Rao et al. [25] and Tanaka et al. [26], who found a high level of COX-2 in colon carcinogen-treated rats at the promotion stage of carcinogenesis. This finding was further supported by Suzuki et al. [27], who found high levels of COX-2 in AOM-induced colonic mucosa. Previous studies have also shown that COX-2 can be upregulated via nuclear β-catenin accumulation, which might be associated with transcriptional regulation by the Wnt signaling pathway [28].

Reduction in the staining intensities for COX-2 was observed in colon tumors from rats treated with brewers’ rice diet (Figure 5), demonstrating the anti-inflammatory property of brewers’ rice. Dietary administration of brewers’ rice has been shown to decrease COX-2 expression in a dose-dependent manner (Table 3). The expression of COX-2 in the AOM-alone group was higher than brewers’ rice-fed groups, but this difference was not significant (P>0.05) (Table 3). This finding was consistent with the results obtained by Roschek et al. [29], which showed that stabilized rice bran extracts inhibited COX-2 activity. This result was also further supported by Norazalina et al. [30], who reported that the expression of COX-2 was weaker in the cytoplasm after treatment with rice bran phytic acid. Several studies have also found that fermented brown rice and rice bran [31], rice bran [32], and germinated brown rice [33] reduced colon carcinogenesis and the expression of COX-2. These results suggested that dietary administration of brewers’ rice reduced colon tumor multiplicity via an anti-inflammatory mechanism involving COX-2 expression. Lower COX-2 expression in rats indicated that brewers’ rice might be associated with the suppression of cell proliferation and induction of apoptosis, which may contribute to a decrease in the number of colon incidences and multiplicities of tumors (adenomas and adenocarcinomas). Thus, the effect of treatment with brewers’ rice on the cell proliferation marker Ki-67 in the AOM-injected rats was examined.

### Effect of brewers’ rice on colorectal cancer cell proliferation

The growth rate of tumors can be determined using the rate of proliferation and death of tumor cells [34]. The most reliable methods to examine colorectal cell proliferation are via the evaluation of Ki-67 using immunostaining and proliferating cell nuclear antigen (PCNA) [35]. Ki-67 is a granular component of the nucleolus, which is expressed exclusively in proliferating cells and is normally used as a cell growth marker [36]. Colorectal cell proliferation, which is reflected by immunohistochemical staining in Ki-67, was compared in the normal, AOM alone, and treatment groups. Colonic tumors in the AOM-alone group showed intense staining for Ki-67 in the nucleus. In contrast, the expression of Ki-67 from the brewers’ rice group was lower, and scattered staining was found in the nucleus (Figure 5). Moreover, Ki-67 expression in the nucleus was also markedly inhibited by treatment with brewers’ rice (Figure 5).

Our data revealed that none of the Ki-67 expression was found in the normal group. Ki-67 expression in colorectal tumor tissue from the AOM-alone group was significantly higher (P<0.05) compared to 20% and 40% (w/w) brewers’ rice. Nevertheless, a reduction in cell proliferation observed in immunohistochemical staining is one of the modes of action in which brewers’ rice is thought to exhibit its chemopreventive efficacy, and a significant reduction in cell proliferation was observed (20% and 40% (w/w) brewers’ rice) (P<0.05), which further support our previous *in vitro* cytotoxicity activity of water extract of brewer’s rice on the colon cancer (HT-29) cell line [13]. However, no significant differences were observed in Ki-67 expression between the AOM-alone group and 10% (w/w) brewers’ rice (P>0.05) (Table 3).

A reduction in tumor incidence is generally correlated to a decrease in cellular proliferation and/or increase in apoptosis [37]. Because brewers’ rice was shown to decrease cell proliferation in a dose-dependent manner compared to AOM alone, the development of this natural product against colorectal cancer may be promising.

### Brewers’ rice induces apoptosis in colonic tumors

Normally, the growth rate of preneoplastic or neoplastic cells is excessive compared to normal cells due to dysregulation of the cell-growth and cell-death machineries [38]. As shown in Table 2, histological evaluation using H&E staining indicated that the majority of the colon tumor incidence and tumor multiplicity were present in the AOM-alone group compared to the brewers’ rice groups. Thus, colon tissue sections were evaluated using TUNEL staining to examine the presence of apoptotic cells. Drug resistance to cancer cells is reflected in its resistance against apoptosis. Thus, the induction of apoptosis is a critical mechanism in the chemoprevention and chemotherapy of cancer [38].

**Figure 6:**
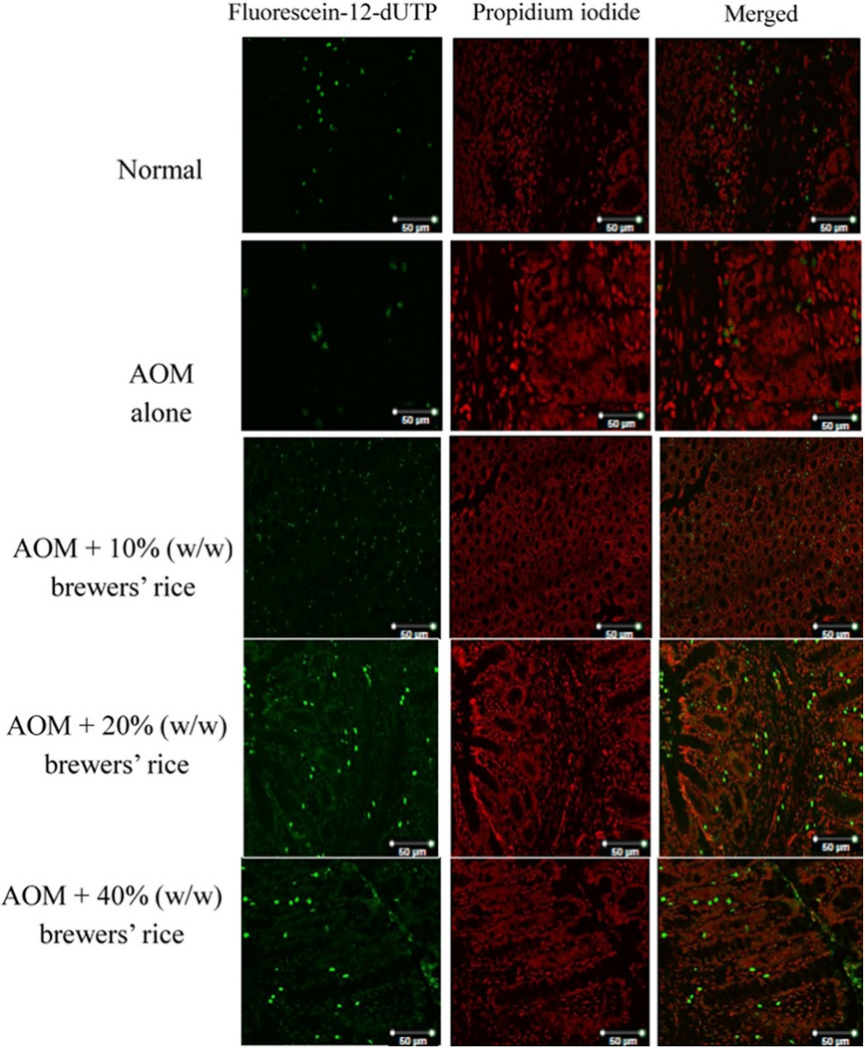
**TUNEL assay of brewers’ rice-treated AOM-induced colon cancer (n = 3).** Cells with green stained nuclei are apoptotic cells (400× magnification).

Using PI as a nuclear counterstain (red), TUNEL staining showed a large number of apoptotic cells (green staining) in brewers’ rice-treated groups compared to the AOM-alone group (Figure 6). In the AOM-alone group, the mean number of apoptotic cells was 4.08%. Analysis of TUNEL staining revealed that the administration of brewers’ rice in AOM-induced rat colorectal cancer resulted in a dose-dependent increase in apoptotic cells as shown by an increase in green fluorescence (Figure 6). However, no significant difference was observed in the apoptotic counts between the AOM alone and group treated with 10% (w/w) brewers’ rice (P>0.05) (Table 4).

**Table 4:** **TUNEL assay for brewers’ rice-treated AOM-induced colon cancer**

Group no.	Treatment	Apoptotic cells (%)	Non-apoptotic cells (%)
1	Normal	4.82 ± 1.89^b^	95.18 ± 1.89^b^
2	AOM alone	4.08 ± 2.30^b^	95.92 ± 2.30^b^
3	AOM + 10% (w/w) of brewers’ rice	6.70 ± 0.99^b^	93.31 ± 0.99^b^
4	AOM + 20% (w/w) of brewers’ rice	20.68 ± 10.92^a^	79.33 ± 10.92^a^
5	AOM + 40% (w/w) of brewers’ rice	31.89 ± 5.22^a^	68.12 ± 5.22^a^

Each value expressed as mean ± SD of three determinations. Value in the same column with different superscript letter indicates significant difference by Tukey test (P<0.05). Administration of 20% and 40% (w/w) of brewers’ rice significantly increased in the apoptotic cells compared to the AOM-alone group (P<0.05). However, the apoptotic cells did not significantly differ between the AOM-alone group and the 10% (w/w) of brewers’ rice (P>0.05).

Taken together, treatment of brewers’ rice resulted in the increase of the apoptotic cells, with a maximum effect marked at 40% (w/w) brewers’ rice. A significant increase in the apoptotic cells was also observed in the group treated with 20% and 40% (w/w) brewers’ rice (P<0.05) compared to the AOM-alone group. This finding indicated that brewers’ rice not only inhibited cell proliferation, but it also induced apoptosis in colon tumor tissue. Thus, this may represent an appropriate approach to suppress the promotion and progression of carcinogenesis via the induction of apoptosis by dietary chemopreventive agents. Moreover, it is plausible that dietary administration of brewers’ rice reduced the colon tumor incidence and multiplicity via inhibition of cell proliferation and induction of apoptosis.

Most of the study primarily concentrated on individual phytochemical effects and their potential effects on the growth inhibition and prevention of cancer; however, the importance of these individual phytochemical might be difficult to confirm [39]. The majority of the data indicated the protective effects of additive and/or synergistic effects of several components [39–41]. Thus, in the present study, brewers’ rice rather than extracts of individual phytochemical components were fed to the rats. We observed a greater reduction on tumor incidence and multiplicity feeding with brewers’ rice. We speculated that this was partially due to the composition of nutrients and bioactive components/phytochemicals, which act synergistically to contribute to this marked reduction of tumor incidence and multiplicity. Dietary chemoprevention studies have revealed that the magnitude of anticancer activity in the whole food or whole food extract is greater than its isolated compounds [42, 43]. Previous studies have also reported that consuming whole rice bran is important for providing comprehensive protection against cancerous cells compared to the protection provided by isolated constituents [10, 31, 44, 45].

Present study demonstrated that the treatment of brewers’ rice resulted in the increase of the apoptotic cells reveals that the observed effects were probably attributed by the dietary constituents present in the brewers’ rice. Data from our previous study demonstrated that brewers’ rice contains phenolic, antioxidant activity, phytic acid, vitamin E, and γ-oryzanol [13]. The synergistic/additive effects of these compounds in the brewers’ rice may lead to the increase of apoptotic effects seen in this study.

## Conclusions

Our study indicated that brewers’ rice reduces colon tumor incidence and multiplicity via the inhibition of proliferation and induction of apoptosis, in which both effects may represent a general property of brewers’ rice. However, further investigations are needed to elucidate the molecular mechanisms that are associated to control and execute apoptotic cell death. Taken together, our data suggested that brewers’ rice can inhibit cell proliferation, induce apoptosis, and suppress COX-2 and β-catenin expression via the Wnt signaling pathway and holds great promise in the field of chemoprevention as a dietary agent.

## Abbreviations

ACF: Aberrant crypt foci
AIN-93G: American institute of nutrition
AOM: Azoxymethane
APES: 3-aminopropyltrimethoxysilane
COX-2: Cyclooxygenase-2
DAB: 3, 3'Diaminobenzidine
H_2_O_2_: Hydrogen peroxide
HT-29: Colorectal cancer
H&E: Hematoxylin and eosin
IHC: Immunohistochemistry
LEF: Lymphocyte enhancer factor
PBST: Phosphate-buffered saline in 0.1% Tween-20
PCNA: Proliferating cell nuclear antigen
PI: Propidium iodide
rTdT: Terminal deoxynucleotidyl transferase, recombinant, enzyme
TCF: T-cell factor
TUNEL: TdT-mediated dUTP nick-end labeling.
